# Peptide-based inhibitors hold great promise as the broad-spectrum agents against coronavirus

**DOI:** 10.3389/fmicb.2022.1093646

**Published:** 2023-01-19

**Authors:** Mingxing Tang, Xin Zhang, Yanhong Huang, Wenxiang Cheng, Jing Qu, Shuiqing Gui, Liang Li, Shuo Li

**Affiliations:** ^1^Shenzhen Institutes of Advanced Technology, Chinese Academy of Sciences, Shenzhen, China; ^2^Department of Otolaryngology, Huazhong University of Science and Technology Union Shenzhen Hospital, Shenzhen, China; ^3^School of Medicine, Southern University of Science and Technology, Shenzhen, China; ^4^Center for Translational Medicine Research and Development, Shenzhen Institutes of Advanced Technology, Chinese Academy of Sciences, Shenzhen, China; ^5^Department of Pathogen Biology, Shenzhen Center for Disease Control and Prevention, Shenzhen, China; ^6^Department of Critical Care Medicine, Shenzhen Second People’s Hospital, The First Affiliated Hospital of Shenzhen University, Shenzhen, China

**Keywords:** SARS-CoV-2, viral infection, spike protein, anti-viral peptides, host protease, host receptors

## Abstract

Severe Acute Respiratory Syndrome Coronavirus (SARS-CoV), Middle East Respiratory Syndrome (MERS), and the recent SARS-CoV-2 are lethal coronaviruses (CoVs) that have caused dreadful epidemic or pandemic in a large region or globally. Infections of human respiratory systems and other important organs by these pathogenic viruses often results in high rates of morbidity and mortality. Efficient anti-viral drugs are needed. Herein, we firstly take SARS-CoV-2 as an example to present the molecular mechanism of CoV infection cycle, including the receptor binding, viral entry, intracellular replication, virion assembly, and release. Then according to their mode of action, we provide a summary of anti-viral peptides that have been reported in peer-reviewed publications. Even though CoVs can rapidly evolve to gain resistance to the conventional small molecule drugs, peptide-based inhibitors targeting various steps of CoV lifecycle remain a promising approach. Peptides can be continuously modified to improve their antiviral efficacy and spectrum along with the emergence of new viral variants.

## Introduction

Coronaviruses are membrane enveloped virus particles, which contain a single-stranded positive-sense ribonucleic acid (RNA) genome and a matrix of RNA-associated capsid proteins (Zhou et al., 2020; Li et al., 2022). Taxonomically, four genera are classified within the *coronaviridae* family, including alpha-, beta-, gamma-, and delta-coronaviruses. Among them, seven alpha-and beta-CoV species have been identified as zoonotic coronaviruses (HCoVs). The highly pathogenic members are Severe Acute Respiratory Syndrome Coronavirus (SARS-CoV), Middle East Respiratory Syndrome Coronavirus (MERS-CoV), and the recently emerged SARS-CoV-2, all of which are capable of causing severe respiratory tract infections and acute respiratory distress syndrome (ARDS). Infections by the intensively pathogenic HCoVs, especially SARS-CoV-2, have been the top concern of public health in recent years. The other HCoVs, HCoV-229E, HCoV-NL63, HCoV-OC43, and HCoV-HKU1 that normally cause mild respiratory illness have circulated within human populations for centuries. Although numerous drugs and vaccines have been developed and applied to combating SARS-CoV-2 or subsequent variants, drug resistance raises great concern (Rawson et al., 2020; Tannock et al., 2020; Kasuga et al., 2021; Şimşek-Yavuz and Komsuoğlu elikyurt, 2021). For example, the SARS-CoV-2 B.1.617.2 (delta) variant can rapidly gain resistance to monoclonal antibody after treatment (Rockett et al., 2022). The more recent B.1.1.529 (Omicron) variant is highly resistant to the majority of existing SARS-CoV-2 neutralizing antibodies (Cao et al., 2022; Hoffmann et al., 2022) as well as mRNA vaccines (Cele et al., 2022; Edara et al., 2022). Therefore, effective broad-spectrum antiviral therapeutics are still needed.

Recent observations indicated that peptides of diverse sources (either natural or synthetic) represent a class of promising antivirals. Peptides are small fragments of proteins typically comprising of 2–50 amino acid residues. These peptides achieve viral inhibition through various modes of actions, including direct binding to virions or host cell-surface receptors, blocking viral entry, interfering enzymatic activity to inhibit intracellular replication, and indirectly modulating immune responses(Schütz et al., 2020; Ghosh and Weinberg, 2021; Heydari et al., 2021). Compared to the conventional small molecule drugs, peptide synthesis can be quickly launched and modified (Vagner et al., 2008; Gao et al., 2018). More importantly, the chemical composition makes peptides highly specific and effective to their targets, even at nanomolar or picomolar concentrations (Cao et al., 2020; Schütz et al., 2020; Heydari et al., 2021; Shah et al., 2022; Yang et al., 2022).

Herein, we take SARS-CoV-2 as an instance to introduce the structural and functional properties of coronaviruses, and the viral infection process. Then, a state-of-the-art overview is provided to summarize recent researches that report the anti-CoV efficacy of peptides and their potentials in clinical use.

## CoV genome structure and viral infection mechanism

### Structural and functional dissection of SARS-CoV-2 genome encoded proteins

**Figure 1:**
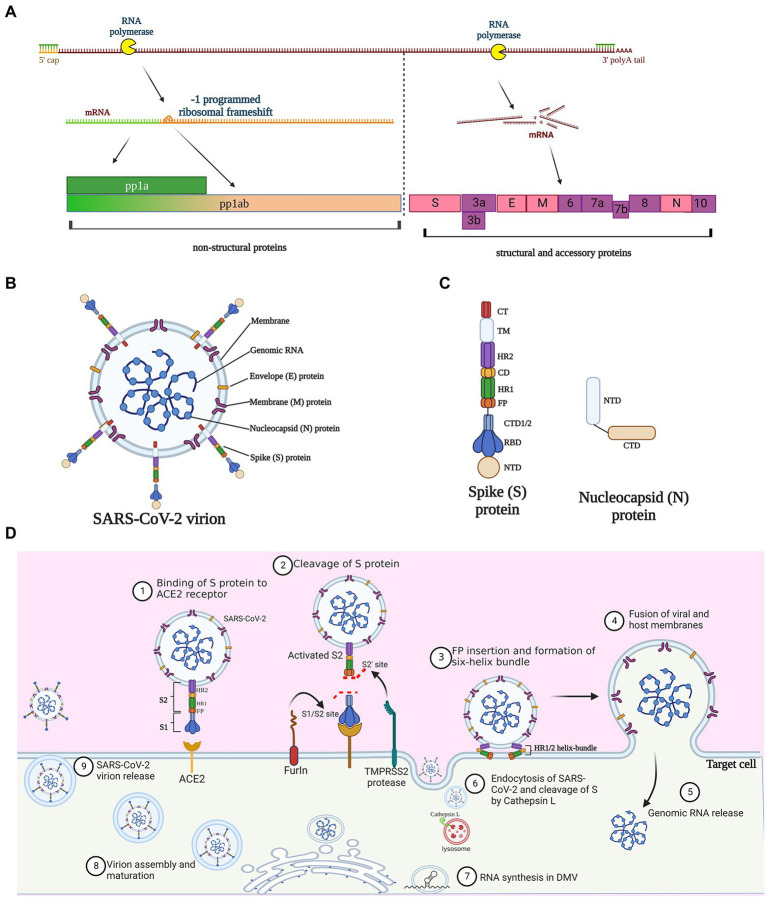
Molecular and structural bases of SARS-CoV-2 infection. **(A)** Proteins encoded by SARS-CoV-2 genome. Three quarters of the genome at the 5′-terminus encode the replicase polyproteins pp1a and pp1ab, which can be cleaved to generate 16 nonstructural proteins; pp1ab is derived from minus 1 site programmed ribosomal frameshift at the stop codon during the synthesis of pp1a. The 3′-terminus one quarter of the viral genome encode four structural proteins and accessory proteins. S, spike; E, envelope; M, membrane; and N, nucleocapsid. **(B)** Schematic diagram of SARS-CoV-2 virion structure. **(C)** Protein structure of spike protein and nucleocapsid protein. NTD, N-terminal domain; RBD, receptor-binding domain; CTD, C-terminal domain; FP, fusion peptide; HR, heptad repeat; CD, connector domain; TM, transmembrane domain; and CT, cytoplasmic tail. **(D)** The infection lifecycle of SARS-CoV-2. TMPRSS2, Type II transmembrane serine protease; ACE2, angiotensin-converting enzyme 2.

The full-length genome of SARS-CoV-2 consists of 29,870 bases with a 5′-cap and a 3′- poly(A) tail of variable length (Wu et al., 2020; Zhu N. et al., 2020; Figure 1A). Three functional types of proteins are encoded by the viral genome (Bai et al., 2022), including (1) structural proteins spike (S), membrane (M), envelop (E), and nucleocapsid (N) that constitute virions; (2) non-structural proteins that are mainly responsible for proteolysis and RNA synthesis; and (3) accessory proteins that are mainly involved in immune evasion (Figure 1B).

The 5′-proximal three quarters of the genome encode the replicases pp1a and pp1ab (Figure 1A), which can be further cleaved by the virus-encoded proteases papain-like protease (PL^pro^) and chymotrypsin-like or main protease (M^pro^), to generate 16 nonstructural proteins (NSP 1–16). The yield balance between pp1a and pp1ab is controlled through a fine-tuning regulatory mechanism named programmed ribosomal frameshifting, which has been nicely summarized elsewhere (Malone et al., 2022). Nonstructural proteins have multiple roles in genome replication, transcription, viral morphogenesis, and dysregulation of host immune responses (Malone et al., 2022; Yan et al., 2022). For example, when mature NSP1 is released from the replicase polyproteins following proteolytic cleavage, it rapidly induces host mRNA degradation and shuts down translation of host proteins (Huang et al., 2011; Thoms et al., 2020), while the other NSPs come to form the replication-transcription complex (RTC). NSP12, in synergy with its auxiliary co-factors NSP7 and NSP8, constitutes the RNA-dependent RNA polymerase complex (RdRp) and serves as the replication/transcription machinery to replicate viral genome, rather than host polymerase (Kirchdoerfer and Ward, 2019; Yan et al., 2021).

The 3′-proximal one quarter of the viral genome is transcribed into a nested set of sub-genomic RNAs that are in turn translated to structural proteins and accessory proteins. As with all CoVs, SARS-CoV-2 structure proteins include S, M, E, and N proteins. The S protein, protruding from the viral surface, binds to the angiotensin-converting enzyme 2 (ACE2) to initiate viral entry into host cells, a vital process for CoV infection (Huang et al., 2020; Walls et al., 2020). Thus, as the most easily accessible but also an indispensable viral component, the S protein has become an attractive target of anti-coronavirus peptides in a vast number of researches (Huang et al., 2020; Schütz et al., 2020). Structurally, S protein possesses two subunits, S1 and S2. The S1 subunit consists of N-and C-terminal domains and an important receptor-binding domain (RBD), while the S2, involved in membrane fusion and viral entry, contains a fusion peptide (FP), two heptapeptide repeat (HR1 and HR2), a transmembrane (TM), and cytoplasmic (CT) domains (Huang et al., 2020; Figure 1C). The E protein is a transmembrane protein responsible for viral assembly, budding, morphogenesis, and trafficking (Schoeman and Fielding, 2019). E protein directly contributes to the viral pathogenesis since it not only activates the host NACHT, LRR, and PYD domain-containing protein 3 (NLRP3) inflammasome (Nieto-Torres et al., 2015), but also undermines the tight junction protein complex of the lung epithelium (Chai et al., 2021; Javorsky et al., 2021). The M protein is the major component of the viral envelop, conferring the virion size and spherical structure. M protein is involved in interaction and trafficking of multiple viral proteins, as well as assembly and release of virion particles (Yan et al., 2022). The SARS-CoV M protein can stimulate the host to produce a specific CD8^+^ T cell immune response (Li et al., 2021). Owning to the high sequence identity (90.5%) of the M protein gene between SARS-CoV-2 and SARS-CoV (Mahtarin et al., 2022), the SARS-CoV-2 M protein is likely to have similar immunogenic effects (Su et al., 2021). The main role of the N protein is binding to genomic RNA to form a ribonucleoprotein complex, which is related to viral replication and assembly (Mcbride et al., 2014; Guo et al., 2016). Compared to the other structure proteins, the gene encoding N protein is highly conserved and stable with few mutations over time (Hodge et al., 2021; Figure 1C). The C-terminal region of N protein favors viral immune evasion by antagonizing the host interferon-beta (IFN-β) pathway (Lu et al., 2011). Given these basic findings, the N protein is a great potential target for diagnosis and therapy against CoV infection.

Eleven genes encoding accessory proteins also locate within the 3′- proximal part of SARS-CoV-2 genome and they are interlaced with structural protein genes. Although the characterization of these accessory proteins is relatively limited, they appear to have important roles in pathogenesis and immune evasion, rather than virus replication (Redondo et al., 2021). Mutations are frequently detected in accessory proteins among variants of concern, indicative of increasing transmissibility and immune evasion (Shang et al., 2020). In light of their frequent mutations, accessory proteins might not be favorable targets of the broad-spectrum anti-coronavirus peptides. Functional analysis of those proteins substantiates the bioinformatic indication. Through diverse strategies, the accessory proteins, ORF3b (Konno et al., 2020), ORF6 (Miorin et al., 2020), ORF7a (Cao et al., 2021), and ORF8 (Lei et al., 2020), can antagonize the type I IFN response, an important host defense reaction against viral infection.

### Infection mechanism of SARS-CoV-2—binding, entry, intracellular replication, virion assembly, and release

The SARS-CoV-2 infection involves multiple steps (Figure 1D). Initially, the S protein is cleaved and activated by the host proprotein convertase furin, leaving the protruding extracellular S1 subunit and the transmembrane S2 subunit non-covalently bounded (Peacock et al., 2021). The cleavage exposes the RBD in S1, which directly interacts with the peptidase domain of ACE2 and induces drastic transformational alteration of S2 (Cai et al., 2020; Liu et al., 2020). The cleavage of S2 by Type II transmembrane serine protease (TMPRSS2) further exposes the fusion peptide, thus facilitating its insertion into cellular membrane (Fraser et al., 2022; Iwata-Yoshikawa et al., 2022). Simultaneously, the HR1 and HR2 in S2 form a six-helix bundle fusion core, which acts as a hinge to bring the viral and host cell membrane in close proximity (Yao H. et al., 2020; Xia et al., 2020b). Alternatively, the pH-dependent enzyme cathepsin L can also implement the cleavage of S2 when viral entry is dependent on endocytosis (Matsuyama et al., 2020; Hoffmann et al., 2020b). After the membrane fusion or endocytosis, the SARS-CoV-2 gRNAs are released into cytosol, and soon translated into two replicase polyproteins pp1a and pp1ab, by hijacking the host cell ribosomes. pp1a and pp1ab are digested by the viral proteases, M^pro^ and PL^pro^, into 16 non-structural proteins, which further form the RTCs for RNA synthesis (Malone et al., 2022). NSP3 and NSP4 drive the rearrangement of the endoplasmic reticulum (ER) into double membrane vesicles (DMVs; Snijder and Limpens, 2020), where the RTCs produce new gRNA and a set of sub-genomic mRNAs that are finally translated into four structural proteins and a few accessory proteins. SARS-CoV-2 assembly commences as the gRNAs are coated with nucleocapsid proteins, resulting in RNA-nucleocapsid complexes that bud into the endoplasmic reticulum-Golgi intermediate compartment (ERGIC) to form mature virions (Boson et al., 2021). Finally, the virus particles are released *via* the budding of the Golgi apparatus and exocytosis of the cell membrane for a new round of infections.

## Peptides working at different infection stages are potent anti-CoV agents

### Peptides targeting initial binding of S protein to the ACE2 receptor

**Figure 2:**
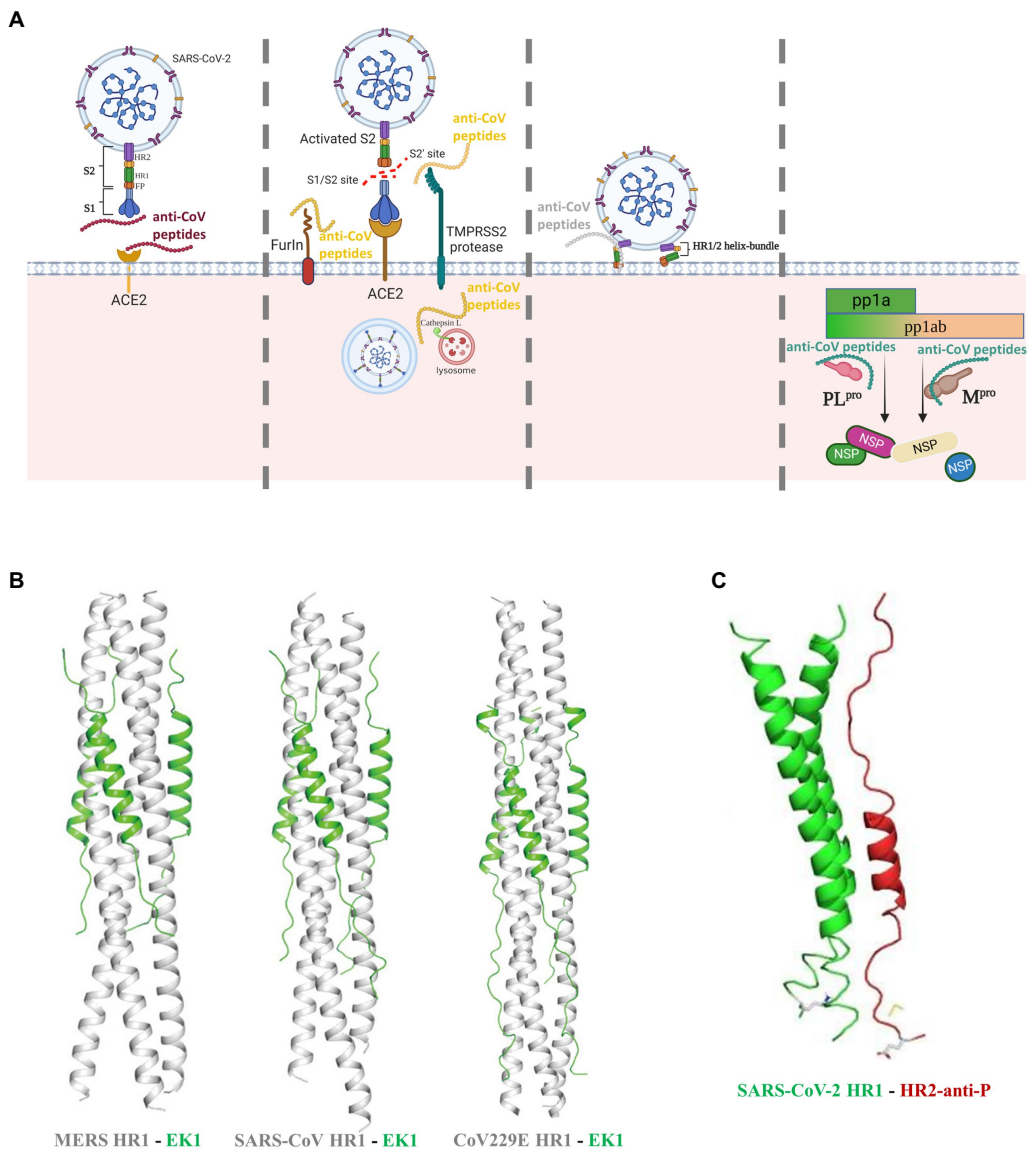
Peptides as potent inhibitors against coronavirus. **(A)** Anti-CoV peptides can work with diverse modes of actions by targeting the binding of S protein to host receptor, or the proteolytic processing of S proteins by host proteases, or the host-viral membrane fusion, or the viral proteases that are responsible for cleavage of replicase polyproteins into non-structural proteins. **(B)** The X-ray crystal structures of membrane-fusion inhibitory peptide EK1 shown as green in complex with the S protein HR1 regions of various CoVs shown as white (Xia et al., 2019b). **(C)** The X-ray crystal structures of membrane-fusion inhibitory peptide HR2-anti-P shown as red binding to the SARS-CoV-2 HR1 region shown as green (Ling et al., 2020).

Targeting the RBD domain in S protein to inhibit its binding to ACE2 has been so far an intensively popular strategy against CoVs (Figure 2A). The charged amino acids between residues 22 and 57 of ACE2 are predicted to be the critical interaction site (Han et al., 2006). In an early work, two peptides P4 and P5 that mimicked this region can bind to SARS-CoV S1 RBD and inhibit pseudo-virion infection with a high half-maximal-inhibitory concentration (IC_50_) of 50 and 6 μM, respectively. Interestingly, another peptide comprised of two discontinuous segments of ACE2 (a.a. 22–44 and 351–357) showed higher antiviral efficacy (IC_50_: 0.1 μM) in a HeLa cell model (Han et al., 2006). The S proteins of SARS-CoV and SARS-CoV-2 share 76% sequence homology while their RBDs share 75% similarity (Jaimes et al., 2020). Although SARS-CoV-2 has greater ACE2 binding affinity (Wrapp et al., 2020) and higher transmissibility (Zhou et al., 2020), the high sequence similarity indicates that peptides effectively blocking the S1 RBD of SARS-CoV might also inhibit SARS-CoV-2 infection. To address the SARS-CoV-2 infection, a series of RBD-targeting peptides have been synthesized or discovered (Cao et al., 2020; Jaiswal and Kumar, 2020; Tavassoly et al., 2020; Wang et al., 2021). Using ACE2 as the scaffold, researchers synthesized two peptides AHB1 and AHB2, which neutralized SARS-CoV-2 with IC_50_ values of 35 and 16 nM, respectively (Cao et al., 2020). Surprisingly, another two peptides (LCB1 and LCB3) based on *de-novo* sequencing of the RBD-binding motifs showed a much higher potency in preventing SARS-CoV-2 infection of mammalian Vero-E6 cells, with IC_50_ values of 23.54 and 48.1 pM, respectively (Cao et al., 2020). Except the abovementioned synthetic peptides, a natural peptide produced by airway epithelium, human cathelicidin LL37, can bind to the S1 RBD and inhibit SARS-CoV-2 S pseudo-virion infection with a IC_50_ value of 4.74 μg/ml (Wang et al., 2021). Notably, the RBD is not the exclusive ACE2-interaction site, since peptides targeting other regions in S1 were also able to neutralize SARS-CoV (Zheng et al., 2005) and, thus potentially SARS-CoV-2.

HCoV-NL63, SARS-CoV, and SARS-CoV-2 employ ACE2 as the entry receptor, thus peptides targeting ACE2 can shield the binding of S proteins (Figure 2A). Based on this, different inhibitors cloaking the ACE2 have been therefore synthesized, but their antiviral efficacy varies considerably, with IC_50_ values ranging from nanomolar to millimolar concentrations (Huang et al., 2003; Hu et al., 2005; Ho et al., 2006; Struck et al., 2012). Of note, recent studies discovered that some natural peptides can also target ACE2 to inhibit SARS-CoV-2 infection (Wang et al., 2020; Beddingfield et al., 2021). Human defensin 5 (HD5), a natural lectin-like α-defensin produced by the Paneth cells, can stably bind to ACE2 and prevent infection of S protein-expressing pseudo-virions at concentrations as low as 10 μg/ml (Wang et al., 2020). ATN-161, a fibronectin derivative, might also bind to ACE2 to significantly reduce SARS-CoV-2 infection (IC_50_: 3 μM; Beddingfield et al., 2021). However, clinical use of such ACE2-blocking peptides or drugs warrants serious attention, as ACE2 belongs to the renin-angiotensiongen system where it promotes vasodilation, while interference might have life-threatening side effects.

### Peptides targeting proteolytic activation of S protein

As mentioned above, cleavage and activation of S proteins by host proteases are crucial to establish CoV infection, while peptides targeting them can efficiently inhibit viral infection (Figure 2A). Although proteins of multiple viruses can be activated by the proprotein convertase furin (Volchkov et al., 1998; Sugrue et al., 2001; Braun and Sauter, 2019), this feature distinguishes SARS-CoV-2 from SARS-CoV (Matsuyama et al., 2018; Schütz et al., 2020). Two recent studies showed that treatment by the furin inhibitors decanoyl-RVKR-chloromethylketone and MI-1851 can abolish furin cleavage and inhibit SARS-CoV-2 infection of mammalian cells (Bestle et al., 2020; Cheng et al., 2020). Different from furin, TMPRSS2 is involved in proteolytic activation of more CoV species (Shen et al., 2017; Böttcher-Friebertshäuser, 2018), while inhibitors blocking its enzymatic activity could be promising broad-spectrum antivirals. A previous work demonstrated that three different TMPRSS2 inhibitors strongly prevented SARS-CoV-2 and SARS-CoV multiplication in Calu-3 cells in a dose-dependent manner (Bestle et al., 2020). More excitingly, Shapira and coworkers recently synthesized a more potent TMPRSS2 inhibitor N-0385, which can act as a pan-SARS-CoV-2 prophylactic and therapeutic agent and inhibit the cellular entry of multiple SARS-CoV-2 variants of concern at nanomolar concentrations (Shapira et al., 2022). Cellular entry of CoVs might occur through either membrane fusion or endocytosis. In the latter case, the S protein should be activated by proteolysis of cathepsin L, a lysosome-associated protease (Gomes et al., 2020; Zhao M. M. et al., 2021). Blocking the cathepsin L to inhibit SARS-CoV-2 has been tested. P9, a derivate peptide of mouse β-defensin-4, exhibited broad antiviral activities against SARS-CoV, SARS-CoV-2, MERS-CoV and influenza virus, *via* interfering cathepsin L and preventing endosomal acidification (Zhao et al., 2016, 2020). In the follow-up works, this peptide was further optimized into P9R and 8P9R, which showed higher level of anti-SARS-CoV-2 potency, with IC_50_ values of 0.9 and 0.3 μg/ml, respectively (Zhao et al., 2020; Zhao H. et al., 2021). In sum, inhibition of host proteases seems a promising antiviral strategy. However, as with the case of ACE2, interference of the physiologically relevant proteases may induce unwanted adverse effect. This calls for sufficient trials in the future to test the potential cytotoxicity and global impact if the protease inhibitors are to be applied to clinical use.

### Peptides targeting membrane fusion process

In the past 2 decades, a vast number of studies have synthesized diverse anti-viral peptides that target the membrane fusion step (Schütz et al., 2020; Heydari et al., 2021). These fusion inhibitors have been so far the most extensively studied and the most promising ones to be translated into therapeutic peptides. Mechanistically, they were designed to mimic one of the HR regions in the S2 subunit, interact with the complementary HR, block formation of the HR1-HR2 helix bundle, and thus interfere with virus-host membrane fusion (Figure 2A). Comparatively, peptides derived from HR1 (that target HR2) appear often poorly active (Bosch et al., 2004; Liu et al., 2004; Xia et al., 2020b), likely owing to their propensity to self-aggregation. A large number of peptides have been synthesized to address the previous SARS-CoV and MERS-CoV pandemics, mostly showing strong anti-CoV activity with IC_50_ values ranging in micromolar concentrations (Barnard et al., 2004; Liu et al., 2004; Yuan et al., 2004; Zheng et al., 2005; Chu et al., 2008; Ujike et al., 2008; O'keefe et al., 2010; Lu et al., 2014; Channappanavar et al., 2015; Zhao et al., 2016; Sun et al., 2017; Wang et al., 2018; Huang et al., 2019; Xia et al., 2019a). Since the HR1 amino acid sequences of SARS-CoV and SARS-CoV-2 share 92.6% similarity and their HR2 is almost identical (Xia et al., 2020b), peptides derived from SARS-CoV HR2 are very likely to inhibit SARS-CoV-2 infection. In agreement with this suggestion, a pan-coronavirus fusion inhibitor EK1, which was previously identified as a potent antiviral agent against SARS-CoV and MERS-CoV (Figure 2B), also reduced SARS-CoV-2 infection of TMPRSS2-negative Vero-E6 cells with an IC_50_ value of 2.5 μM (Xia et al., 2019b, 2020a). Of interest, the IC_50_ value was 10-fold lower in TMPRSS2-positive Caco-2 cells (Conzelmann et al., 2020; Xia et al., 2020a), reinforcing again the importance of S protein processing in CoV infection. Computational analysis is a powerful tool in designing the potent SARS-CoV-2 inhibitors. For instance, at the onset of the COVID-19 pandemic, researchers used the *in silico* approaches to design a potent HR1-targeting peptide to prevent membrane fusion (Figure 2C; Ling et al., 2020). In another work, two potent peptides Fp13-HR1 and Fp14-HR1, that were screened from 17 SARS-CoV HR2-derived fusion inhibitors, were predicted to have a high binding affinity to SARS-CoV-2 HR1, thus they might be effective fusion inhibitors of SARS-CoV-2 (Efaz et al., 2021). To improve antiviral efficacy and stability, rational modifications of the existing inhibitory peptides are also of great importance. This has been nicely exemplified by the modification of the abovementioned EK1 into EK1C4 by linking a cholesterol group to the C-terminus. The optimized peptide displayed more than 19–190-fold potency in preventing the infection of several CoVs, including SARS-CoV-2 (Xia et al., 2020a). Another representative is IPB-02, which was modified from the HR1-targeting peptide IPB-01 by conjugation of a cholesterol group. The refined peptide showed stronger antiviral effect against SARS-CoV-2 with the IC_50_ value decreasing from 22 to 0.08 μM (Zhu Y. et al., 2020). Optimization of fusion inhibitors is not limited to linkage of current peptides to functional groups. Although previously reported HR1-derived peptide inhibitors exhibit poor inhibitory activities, foldon-mediated trimerization of the C-terminus conferred a HR1-derived peptide with higher inhibitory activity against SARS-CoV-2, SARS-CoV-2 variants of concern (VOCs), SARS-CoV, and MERS-CoV (Bi et al., 2022). Moreover, an inspirational work identified that the extended N-terminus of HR2 also involves in interacting with HR1, and a synthetic peptide including this region achieved single-digit nanomolar inhibition of several SARS-CoV-2 variants (Yang et al., 2022).

### Peptides targeting intracellular replication and assembly of coronavirus

Coronavirus infection can also be impeded intracellularly. For example, several chemical compounds Boceprevir, GC-376, calpain inhibitors II and XII had a wide range antiviral activity, *via* a dual mechanism of action by targeting both viral M^pro^ and host cell cathepsin L (Fu et al., 2020; Hu et al., 2020; Ma et al., 2020). These findings indicated that the viral components necessary for RNA replication and assembly are also favorable targets for the anti-CoV peptide design (Figure 2A). Indeed, an early study showed that a M^pro^-targeting octapeptide impeded replication of the SARS-CoV at the concentration of 1 mg·L^−1^ (Gan et al., 2006). Another research reported that Cbz-AVLQ-CN, a broad-spectrum peptide, effectively inhibited six different CoV species with IC_50_ values of 1.3–4.6 μM (Chuck et al., 2014). Interestingly, several active peptides that can bind to both M^pro^ and monoamine oxidase A of SARS-CoV-2 can be generated from hydrolysis of fish proteins, representing potential inhibitors from food source against CoV (Yao Y. et al., 2020). Blocking other enzymes of CoVs is an alternative strategy. Two synthetic peptides K29 and K12 could markedly inhibit the activity of SARS-CoV nsp16 (methytransferase) in a dose-dependent manner, thus disrupting its role in viral RNA synthesis, but viral inhibition assays are still needed (Ke et al., 2012).

In addition to S protein, the N protein might also be a rational target for the anti-CoV peptide design. The C-terminal domains (CTDs) of the N proteins mediate the self-association of the protein to form high-order oligomers, and deletion of 13 amino acids in the HCoV-229E N protein CTD appeared incapable of forming a high degree of oligomerization (Chang et al., 2005). In line with this notion, a C-terminal tail peptide N377–389 interfered with the oligomerization of the CTD of HCoV-229E N protein and inhibited viral replication at 300 μM (Lo et al., 2013). This finding provides insights that blocking the formation of the N protein-RNA higher-order oligomers and in turn the virion assembly can contribute to viral inhibition.

## Perspectives and conclusion

To conclude, peptides that can target various steps in CoV lifecycle have shown great potential in combating CoV infection. In some cases, the peptide-based inhibitors seem to have lower possibility to cause drug resistance (Zhao et al., 2020), and they rarely induce detectable cytotoxicity (Gan et al., 2006). Some peptides exhibit significantly strong and broad-spectrum effect against multiple CoV species. More importantly, combination of peptides with different antiviral mechanisms could generate synergistic impact (Bestle et al., 2020; Hoffmann et al., 2020a). However, to translate the peptides into clinical therapeutics, they should be safe and stable *in vivo*, while many works need more effort on this aspect. Therefore, a future perspective is to refine current peptides to be more effective and long-lasting, as with the case of EK14C, which was subject to two rounds of optimization from OC43-HR2P. To this end, a database containing comprehensive and precise information of 214 unique anti-CoV peptides would contribute to more rational design or modification (Zhang et al., 2022).

## Author contributions

MT, XZ, YH, and LL devised the framework, wrote and revised the manuscript. WC, JQ, SG, SL, LL, and MT contributed to literature search and gave insightful suggestions in revising this work. All authors contributed to the article and approved the submitted version.

## Funding

This work was supported by the National Natural Science Foundation of  China (81900071), the Fellowship of China Postdoctoral Science Foundation (2022M713287), the Medical Research Foundation of Guangdong Province (A2022046), and the Shenzhen Science and Technology Innovation Commission for Research and Development Projects (JSGG20200807171603039, JSGG20191118161401741, CYJ20210324112607020 and zJCYJ20220530141616037).

## Conflict of interest

The authors declare that the research was conducted in the absence of any commercial or financial relationships that could be construed as a potential conflict of interest.

## Publisher’s note

All claims expressed in this article are solely those of the authors and do not necessarily represent those of their affiliated organizations, or those of the publisher, the editors and the reviewers. Any product that may be evaluated in this article, or claim that may be made by its manufacturer, is not guaranteed or endorsed by the publisher.
